# Case Report: Non-traumatic recurrent hemarthrosis after total knee arthroplasty with literature review

**DOI:** 10.3389/fsurg.2026.1731520

**Published:** 2026-04-10

**Authors:** Wencheng Liu, Xiangjie Huang, Rifu Liu

**Affiliations:** Department of Joint Surgery, Yantai Hospital of Wendeng Orthopaedics & Traumatology, Yantai, Shandong, China

**Keywords:** arterial embolization, pseudoaneurysm, recurrent hemarthrosis, synovectomy, total knee arthroplasty

## Abstract

**Background:**

Recurrent hemarthrosis following total knee arthroplasty (TKA) is an uncommon but challenging complication. While angiographic intervention is typically effective, some cases require surgical intervention.

**Case presentation:**

We report a 69-year-old woman who developed recurrent, non-traumatic hemarthrosis of the left knee 11 months after bilateral total knee arthroplasty. Diagnostic digital subtraction angiography (DSA) demonstrated an abnormal angiographic blush in the suspected branch of the lateral inferior genicular artery, raising suspicion of a possible pseudoaneurysm but without definitive diagnostic features. No active contrast extravasation was observed during angiography, and therefore transcatheter arterial embolization was not performed at that time. Surgical exploration revealed synovial hyperplasia, flexion instability, and pulsatile bleeding from a small artery at the posterolateral superior aspect of the knee joint. Ligation, synovectomy, and spacer exchange were performed.

**Outcome:**

The patient experienced complete symptom resolution and restored joint function during a two-year follow-up.

**Conclusion:**

This case underscores that recurrent hemarthrosis after total knee arthroplasty may be multifactorial. In the setting of negative angiography or inability to perform embolization, surgical exploration plays a critical role in identifying occult bleeding sources, mechanical instability, and soft-tissue pathology.

## Introduction

Non-traumatic recurrent hemarthrosis after total knee arthroplasty (TKA) is a rare but significant complication. Studies have identified intra-articular bleeding as a key contributor to aseptic pain following joint replacement surgery (1). Recurrent bleeding after TKA may involve various periarticular vessels, most commonly the popliteal artery, anterior tibial artery, or branches of the genicular arterial system. Given the complex anatomy of the genicular arterial network, which comprises multiple named branches, comprehensive angiographic evaluation is often required to accurately identify the bleeding source (2). Selective angiography and arterial embolization are widely recognized as effective treatments for recurrent hematomas after TKA. However, recurrent bleeding associated with joint instability has been reported only in a limited number of studies and has not been explicitly emphasized as a distinct clinical mechanism. Although angiography and selective embolization are widely accepted as first-line approaches for recurrent hemarthrosis, they may not adequately address concomitant mechanical factors such as joint instability or intra-articular soft-tissue pathology. This case underscores the importance of reassessing biomechanical contributors when symptoms persist after angiographic evaluation, which may help guide more appropriate and timely surgical intervention. This report presents a clinically relevant case of recurrent hemarthrosis after TKA that highlights limitations in current diagnostic and management strategies. This case report was prepared in accordance with the CARE (CAse REport) guidelines (3).

## Case presentation

**Figure 1:**
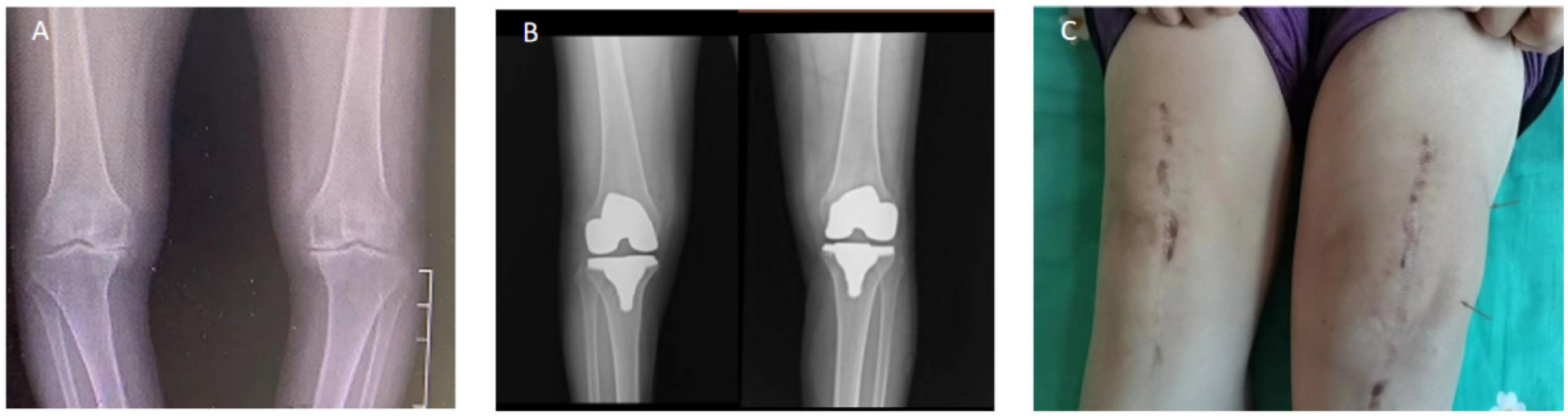
Radiographic and clinical presentation of the patient before and after total knee arthroplasty. **(A)** Preoperative standing anteroposterior radiograph showing severe bilateral knee osteoarthritis with varus deformity. **(B)** Postoperative radiograph after bilateral total knee arthroplasty demonstrating appropriate prosthesis alignment and positioning. **(C)** Clinical image showing significant swelling of the left knee approximately 11 months after surgery.

A timeline summarizing the key clinical events is provided in Table 1. A 69-year-old woman with a history of hypertension and normal coagulation function presented with bilateral knee osteoarthritis and varus deformity (Figure 1A). She underwent bilateral TKA, and postoperative radiographs showed well-positioned prostheses (Figure 1B). The initial recovery was uneventful. Laboratory investigation at presentation showed a normal coagulation profile, with a prothrombin time (PT) of 11.7 s, activated partial thromboplastin time (APTT) of 27.7 s, and an international normalized ratio (INR) of 0.99, ruling out a systemic coagulopathy. She had completed a standard 2-week course of prophylactic anticoagulation (e.g., rivaroxaban) following her index TKA and was not on any antiplatelet or anticoagulant medications at the time of recurrent hemarthrosis.

**Table 1:** Timeline of key clinical events.

Time point (Post-TKA)	Clinical event
0 months	Bilateral TKA performed. Uneventful initial recovery.
11 months	Onset: Left knee swelling and pain after ambulation. Symptoms were intermittent, worsening over subsequent months. According to the patient's medical history, joint aspiration at a local hospital confirmed hemarthrosis (bloody synovial fluid).
18 months	Conservative Treatment Phase: Persistence of severe pain and swelling, leading to inability to walk. Angiography: Digital subtraction angiography performed, revealing abnormal contrast agent development in the posterosuperior region of the femoral prosthesis. No active extravasation seen. During the subsequent period of conservative treatment, the symptoms continued to recur.
24–26 months	Continued Conservative Management: During these two months of conservative treatment, the symptoms progressively worsened.
∼26 months	Surgical Resection: Open surgical exploration performed. Findings: synovial hyperplasia, organized hematoma, and active bleeding. Procedures: Ligation of the pulsatile bleeding artery, thorough synovectomy, and exchange of tibial polyethylene insert (11 mm to 13 mm) to address flexion instability.
26 months – 2 years	Follow-up: Gradual recovery postoperatively.
2 years (final follow-up)	Excellent outcome: Symptom-free, full weight-bearing, restored knee stability and range of motion.

Eleven months postoperatively, the patient developed swelling and pain in the left knee following ambulation, accompanied by difficulty bearing weight. Upon presentation, she was initially managed with a conservative protocol encompassing: (1) rest, cryotherapy, and compressive dressing; (2) a short course of intravenous tranexamic acid; and (3) oral anti-edema medication (e.g., diosmin). The symptoms showed transient improvement with rest but subsequently recurred and progressively worsened over the following months. A previous joint aspiration performed at a local hospital had revealed bloody synovial fluid. By 16 months post-surgery, the patient was unable to walk due to persistent pain and joint swelling (Figure 1C).

At approximately 18 months post-TKA, digital subtraction angiography (DSA) was performed at an outside hospital. The procedural note indicated selective catheterization of the distal left femoral artery, followed by dedicated anteroposterior and lateral angiographic views of the knee joint. Angiography demonstrated an abnormal vascular blush near the posterior aspect of the femoral component, a nonspecific finding that raised suspicion for a possible pseudoaneurysm in the region of a branch of the lateral inferior genicular artery, but without definitive angiographic features.

**Figure 2:**
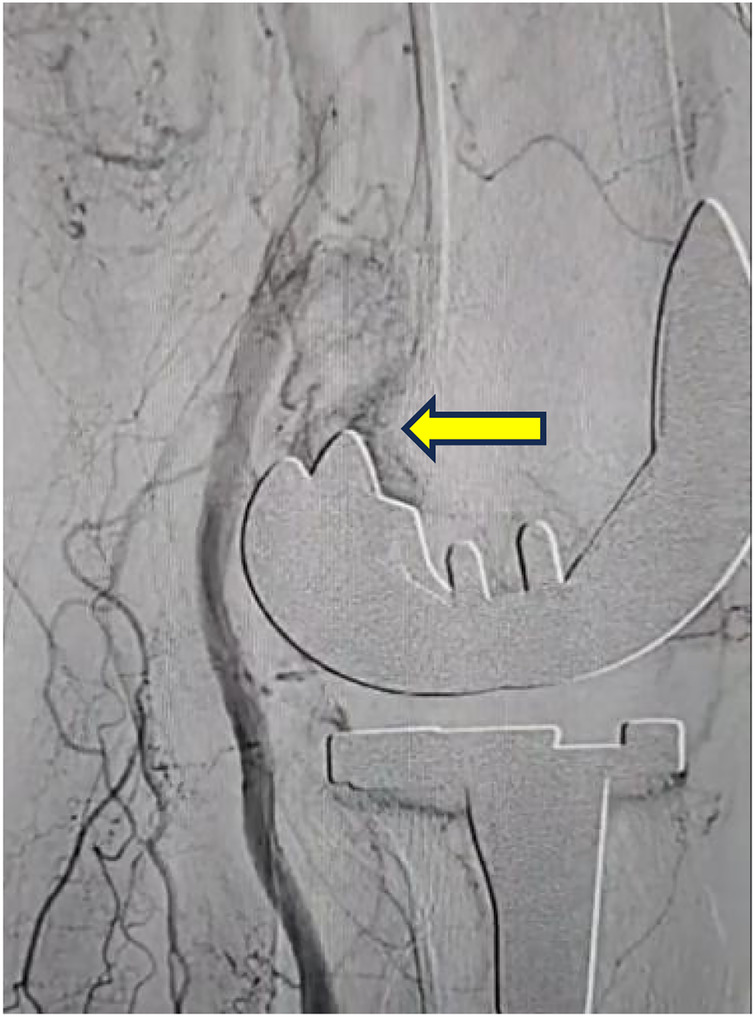
DSA showing abnormal contrast agent development in the posterosuperior region of the femoral prosthesis.

**Figure 3:**
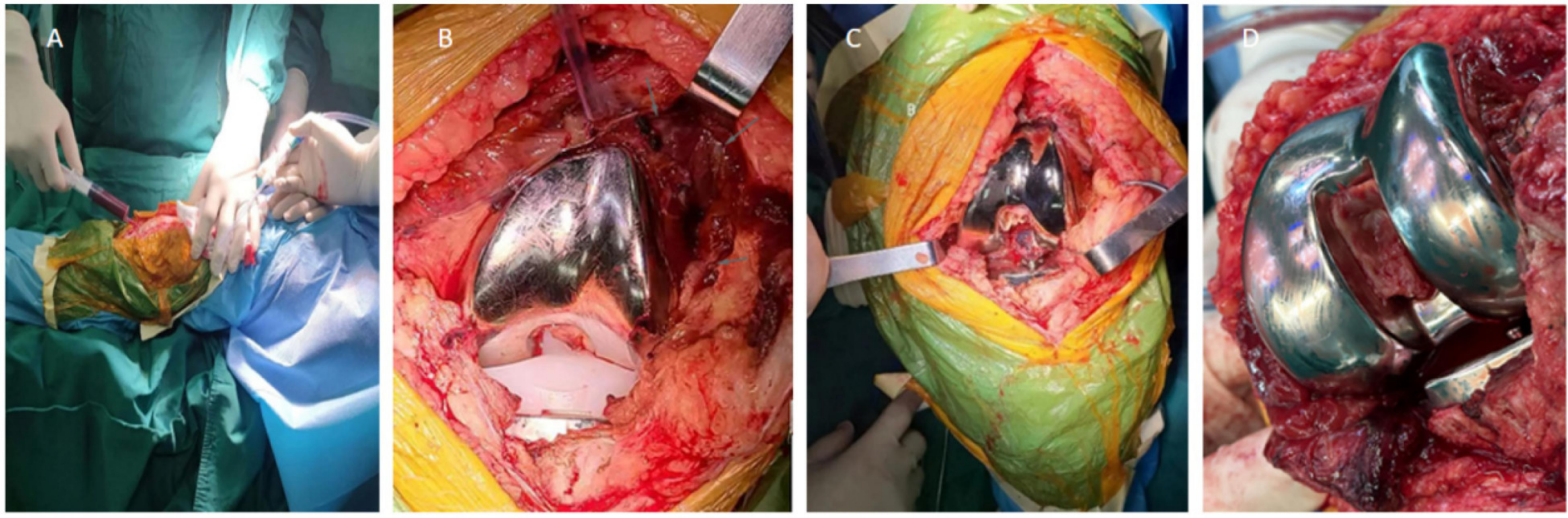
Intraoperative findings: synovial and hematoma pathology. **(A)** Intraoperative aspiration of bloody joint fluid prior to arthrotomy, confirming active hemarthrosis. **(B)** Synovial hyperplasia observed intraoperatively. **(C)** Purplish-red inflamed tissue surrounding the joint capsule. **(D)** Clotted hematoma in the posterior compartment of the knee.

**Figure 4:**
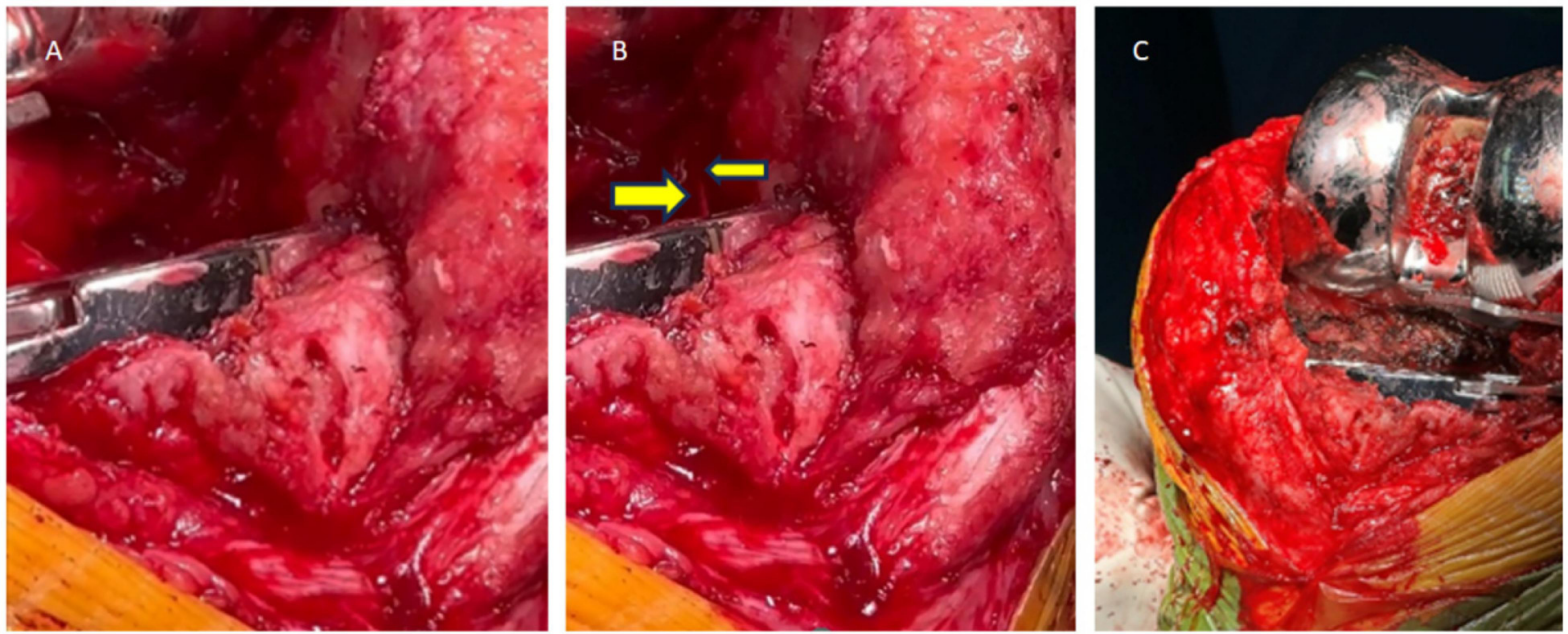
Intraoperative findings: pulsatile bleeding vessel and status post hemostasis. **(A, B)** Intraoperative views of the posterolateral capsular region of the knee, in which **(A)** shows the initial surgical exposure and **(B)** shows the same operative field with active, pulsatile bleeding from a small vessel indicated by arrows, suggestive of a suspected branch of the lateral inferior genicular artery. **(C)** Intraoperative image following spacer removal, ligation, and electrocautery; no further bleeding observed after tourniquet release.

Notably, no active contrast extravasation was visualized. According to the available records, transcatheter arterial embolization was not performed following this diagnostic angiogram (Figure 2). As conservative management failed to resolve the symptoms, and the swelling and pain progressively worsened during the two months preceding surgery, the patient ultimately underwent open surgical exploration approximately 26 months after TKA. Intraoperative aspiration of bloody joint fluid prior to arthrotomy confirmed active hemarthrosis (Figure 3A). Intraoperatively, extensive hyperplastic and inflamed synovial tissue was observed around the posterolateral joint capsule (Figures 3B, C), along with organized hematoma (Figure 3D). A small vessel with pulsatile bleeding in the posterolateral region of the femoral component was identified as the source of active pulsatile bleeding (Figures 4A, B). Given the potential for such proliferative synovium to cause recurrent bleeding through mechanical irritation or impingement, a thorough synovectomy was performed. After achieving hemostasis, knee stability was assessed intraoperatively under anesthesia. Manual varus–valgus stress testing was performed and revealed increased lateral laxity of the affected knee compared with the contralateral side. Manual stress testing revealed lateral joint laxity in both extension and flexion. With the original polyethylene insert in place, the varus–valgus gap measured approximately 4 mm at 30° of knee flexion, while anterior tibial translation was approximately 5 mm at 90° of flexion, accompanied by about 3° of hyperextension, indicating mild flexion–extension instability. Trial reduction with a 13-mm tibial polyethylene insert significantly improved stability compared to the original 11-mm insert. Because the collateral ligaments were intact and the prosthetic components were well fixed, isolated polyethylene insert exchange was considered an appropriate surgical strategy to restore joint stability, as previously reported for the management of prosthetic knee instability after total knee arthroplasty (4, 5). Although the retrieved 11-mm insert showed signs of mild polishing, the primary indication for its exchange was to address joint instability. Consequently, vascular ligation and thorough synovectomy were performed. In addition, the tibial polyethylene insert was exchanged from 11 mm to 13 mm to address flexion instability. After hemostasis and tourniquet release, no recurrent bleeding was observed (Figure 4C).

## Outcome and follow-up

The patient's recovery was uneventful. At the two-year follow-up, objective assessment demonstrated favorable clinical outcomes:

Range of Motion: The left knee achieved full extension (0°) with a flexion arc of 125°, a significant improvement from the preoperative status (10°–100° of flexion with a −10° flexion contracture) and the painful, swollen condition prior to surgery.

Stability: Postoperative physical examination revealed a stable knee with no detectable laxity on varus/valgus stress testing. This corresponded to a perfect score of 25/25 on the stability subscale of the Knee Society Score (KSS), compared to the instability observed under anesthesia preoperatively (medial/lateral joint line opening >2 mm).

Functional Status: The patient regained full, pain-free weight-bearing capacity. She could walk independently without any assistive devices over distances exceeding 1 km and could ascend and descend stairs without difficulty. Her overall functional capacity, as measured by the KSS Functional Score, improved to 90 out of 100.

**Figure 5:**
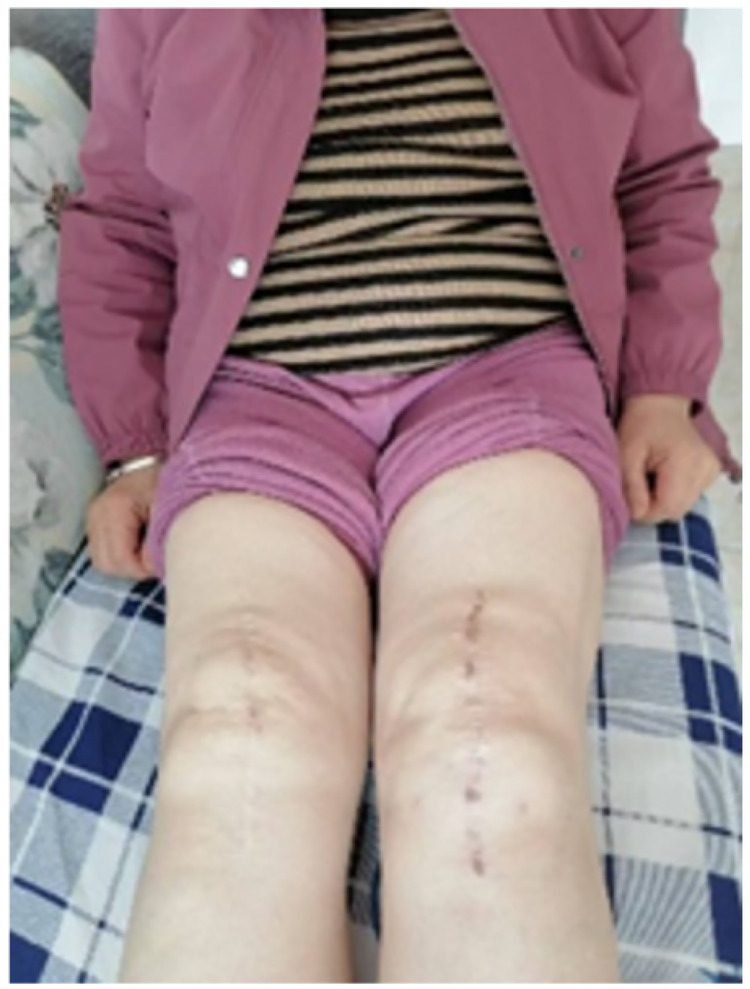
Clinical appearance at the 2-year follow-up. Comparison with Figure 1C shows complete resolution of left knee swelling, with restoration of normal periarticular contours.

Clinical and Radiographic Appearance: Compared to the pronounced swelling present at the time of presentation (Figure 1C), the clinical photograph at two years demonstrated complete resolution of soft-tissue swelling and restoration of normal knee contour (Figure 5).

Patient-Reported Outcome: The patient expressed complete satisfaction with the surgical outcome and had resumed all activities of daily living without limitation.

## Discussion

Recurrent hemarthrosis (RH) after TKA is a rare but disabling complication, with reported incidence ranging from 0.1% to 1.6 % (6). The etiology is multifactorial, involving systemic conditions [e.g., anticoagulation (7), coagulopathies (8–10)], local pathology [e.g., synovial hypertrophy (11, 12) which may lead to impingement (13)], and vascular anomalies such as pseudoaneurysms and arteriovenous malformations (14–16). In the present case, systemic causes such as coagulation disorders or ongoing anticoagulant therapy were considered unlikely, as coagulation parameters were within normal ranges and no antiplatelet or anticoagulant medications were being used at the time of symptom onset. In many cases, the exact cause is elusive, necessitating a stepwise diagnostic and therapeutic approach.

Management of RH typically begins with conservative measures, including joint rest, cryotherapy, compression, or intra-articular thrombin injection (17). For persistent or recurrent cases, diagnostic DSA is pivotal to identify vascular sources, and subsequent transcatheter arterial embolization (TAE) has emerged as the preferred minimally invasive therapeutic option, demonstrating success rates of 80%–90% in recent series (18, 19). However, the decision to proceed with TAE depends on clear identification of a culprit vessel and the technical feasibility of safe embolization (20, 21).

The present case offers an important nuance to this paradigm. Although DSA identified a suspicious vascular blush in the region of a suspected branch of the lateral inferior genicular artery, the absence of active contrast extravasation at the time of angiography, which may be related to intermittent bleeding, as well as anatomical constraints such as vessel overlap or prosthesis-related artifacts, may have contributed to the interventionalist's decision not to perform embolization. This observation underscores that a positive DSA finding does not necessarily mandate or guarantee successful embolization. Importantly, recurrent hemarthrosis after total knee arthroplasty is often multifactorial, with vascular, mechanical, and soft-tissue factors potentially coexisting. When endovascular intervention is not performed or proves ineffective and symptoms persist, open surgical exploration becomes a critical and definitive management step (22).

The present case highlights the importance of recognizing mechanical instability and synovial impingement as coexisting factors in RH. Although angiography showed abnormal contrast agent development in the posterosuperior region of the femoral prosthesis, the patient's symptoms persisted in the absence of definitive endovascular treatment. Intraoperative findings confirmed pulsatile bleeding from the affected vessel and extensive synovial hyperplasia. While direct, dynamic impingement was not captured intraoperatively, the presence of substantial synovial hyperplasia presented a clear rationale for synovectomy to eliminate a potential source of mechanical irritation and recurrent micro-bleeding, as supported by the literature (11, 12). Hemostasis was achieved via vascular ligation and synovectomy, and polyethylene insert exchange restored joint stability. These findings suggest that RH may result from a combination of vascular lesions and biomechanical imbalance, both of which require targeted treatment.

Joint instability has been identified as a significant contributing factor to intra-articular hemorrhagic effusion after total knee arthroplasty (13). Clinicians should be aware that recurrent hemarthrosis after TKA, especially when not amenable to or failing angiographic management, may signal underlying joint instability or soft-tissue pathology. Although vascular abnormalities are most commonly reported, previous studies have suggested that mechanical factors, including prosthetic instability or synovial impingement, may account for a subset of recurrent hemarthrosis cases following TKA (22, 23). Based on the present case, a pragmatic diagnostic-therapeutic approach may be considered. Initial evaluation should include exclusion of systemic causes and angiographic assessment to identify potential vascular lesions, with selective embolization performed when a clear bleeding source is identified. Persistent or recurrent symptoms despite angiographic evaluation should prompt reassessment for mechanical factors, such as joint instability or synovial pathology. Crucially, as demonstrated in this case, flexion-extension instability may be detectable only under general anesthesia—a finding that is both clinically significant and diagnostic. It reveals a “compensated instability” masked by muscular guarding during conscious examination (24), which serves as the direct mechanical substrate for recurrent hemarthrosis (13, 25). We acknowledge that, due to factors such as prosthesis-related imaging artifacts, overlapping vascular territories, and differences in knee position and viewing angle, the angiographic blush may appear not to correspond exactly to the intraoperative bleeding point, even when both findings originate from the same general peri-prosthetic region (26, 27). In this context, increasing the polyethylene insert thickness may be considered an appropriate intervention in selected cases to correct the underlying instability (28). Therefore, examination under anesthesia (EUA) may be considered a valuable diagnostic tool when mechanical etiology is suspected despite negative conventional workup (29). In such situations, early surgical exploration may be warranted, and treatment response should be judged by symptom resolution, restoration of stability, and functional recovery rather than imaging findings alone. A thorough assessment combining imaging, physical examination, and intraoperative exploration is essential. This case reinforces the role of open surgery as an effective treatment for complex RH, especially when multiple etiologic factors are involved (12, 30). In the present case, intraoperative assessment under anesthesia uncovered significant flexion-extension instability that was not apparent during preoperative examination. This instability, addressed by upsizing the polyethylene insert, is hypothesized to have acted as a persistent biomechanical irritant. The abnormal shear and tensile forces on the periarticular tissues, particularly on the already compromised vessel wall and the hyperplastic synovium, likely created a cycle of recurrent micro-trauma and bleeding. This aligns with the understanding that mechanical factors, such as impingement or instability, can coexist with and exacerbate vascular pathologies, leading to refractory hemarthrosis (12, 13).These findings underscore the importance of evaluating both vascular and biomechanical factors in patients with recurrent hemarthrosis after TKA, especially when initial interventions fail.

Nevertheless, several limitations should be acknowledged. First, this study represents a single case report, and therefore the findings may not be generalizable to all patients with recurrent hemarthrosis after total knee arthroplasty. Second, the initial digital subtraction angiography was performed at an outside institution, and detailed procedural information regarding the decision not to perform embolization was unavailable. Consequently, the precise rationale for the interventional management strategy could not be fully verified. Future studies involving larger case series are warranted to further clarify the interaction between vascular lesions and biomechanical instability in the pathogenesis of recurrent hemarthrosis after TKA.

## Conclusion

This case illustrates that recurrent hemarthrosis following total knee arthroplasty may result from a combination of vascular lesions and mechanical instability. When angiography fails to identify a definite treatment target or embolization is not feasible, open surgical exploration can be both diagnostic and therapeutic. Identifying and addressing underlying biomechanical factors, such as synovial impingement and flexion–extension instability, is essential for achieving durable symptom resolution. A multidisciplinary and individualized approach remains critical for managing complex cases of post-TKA hemarthrosis.

## Data Availability

The original contributions presented in the study are included in the article/Supplementary Material, further inquiries can be directed to the corresponding authors.
